# Fasting glucose and body mass index as predictors of activity in breast cancer patients treated with everolimus-exemestane: The EverExt study

**DOI:** 10.1038/s41598-017-10061-2

**Published:** 2017-09-06

**Authors:** Laura Pizzuti, Paolo Marchetti, Clara Natoli, Teresa Gamucci, Daniele Santini, Angelo Fedele Scinto, Laura Iezzi, Lucia Mentuccia, Loretta D’Onofrio, Andrea Botticelli, Luca Moscetti, Francesca Sperati, Claudio Botti, Francesca Ferranti, Simonetta Buglioni, Giuseppe Sanguineti, Simona Di Filippo, Luigi di Lauro, Domenico Sergi, Teresa Catenaro, Silverio Tomao, Antonio Giordano, Marcello Maugeri-Saccà, Maddalena Barba, Patrizia Vici

**Affiliations:** 1 Division of Medical Oncology 2, Regina Elena National Cancer Institute, Via Elio Chianesi 53, 00144 Rome, Italy; 2 Medical Oncology Unit Policlinico Sant’Andrea, Via di Grottarossa 1035/1039, 00189 Rome, Italy; 3 Department of Medical, Oral and Biotechnological Sciences and CeSI-MeT, G. d’Annunzio University, Via dei Vestini, 66100 Chieti, Italy; 4 Medical Oncology Unit, SS Trinità Hospital, S.Marciano, 03039 Sora, Frosinone Italy; 5 Department of Medical Oncology, Campus Bio-Medico University of Rome, Via Álvaro del Portillo, 200, 00128 Roma, Italy; 6 Medical Oncology, Ospedale San Giovanni Calibita Fatebenefratelli, ISOLA TIBERINA, Piazza In Piscinula 13 -, 00153 Roma, Italy; 7 Department of Oncology and Haematology, Azienda Ospedaliera Policlinico, Via del Pozzo 71, 41124 Modena, Italy; 8 Biostatistics Unit, Regina Elena National Cancer Institute, Via Elio Chianesi 53, 00144 Rome, Italy; 9 Department of Surgery, Regina Elena National Cancer Institute, Via Elio Chianesi 53, 00144 Rome, Italy; 10 Department of Radiology, Regina Elena National Cancer Institue, Via Elio Chianesi 53, 00144 Rome, Italy; 11 Department of Pathology, Regina Elena National Cancer Institute, Via Elio Chianesi 53, 00144 Rome, Italy; 12 Department of Radiation Oncology, Regina Elena National Cancer Institute, Via Elio Chianesi 53, 00144 Rome, Italy; 13 Emergency Department, Santa Maria Goretti Hospital, Via Canova 3, 04100 Latina, Italy; 14 Department of Medico-Surgical Sciences and Biotechnologies, La “Sapienza” University of Rome, Oncology Unit, Istituto Chirurgico Ortopedico Traumatologico, Via Franco Faggiana 1668, 04100 Latina, Italy; 15 Sbarro Institute for Cancer Research and Molecular Medicine e del Center for Biotechnology, College of Science and Technology, 1900 N, 12th Street, Temple University, Philadelphia, PA USA; 16 Scientific Direction, Regina Elena National Cancer Institute, Via Elio Chianesi 53, 00144 Rome, Italy

## Abstract

Evidence on everolimus in breast cancer has placed hyperglycemia among the most common high grade adverse events. Anthropometrics and biomarkers of glucose metabolism were investigated in a observational study of 102 postmenopausal, HR + HER2- metastatic breast cancer patients treated with everolimus-exemestane in first and subsequent lines. Best overall response (BR) and clinical benefit rate (CBR) were assessed across subgroups defined upon fasting glucose (FG) and body mass index (BMI). Survival was estimated by Kaplan-Meier method and log-rank test. Survival predictors were tested in Cox models. Median follow up was 12.4 months (1.0–41.0). The overall cohort showed increasing levels of FG and decreasing BMI (p < 0.001). Lower FG fasting glucose at BR was more commonly associated with C/PR or SD compared with PD (p < 0.001). We also observed a somewhat higher BMI associated with better response (p = 0.052). More patients in the lowest FG category achieved clinical benefit compared to the highest (p < 0.001), while no relevant differences emerged for BMI. Fasting glucose at re-assessment was also predictive of PFS (p = 0.037), as confirmed in models including BMI and line of therapy (p = 0.049). Treatment discontinuation was significantly associated with changes in FG (p = 0.014). Further research is warranted to corroborate these findings and clarify the underlying mechanisms.

## Introduction

The mammalian target of rapamycin (mTOR) inhibitor everolimus has been approved in combination with exemestane for treatment of hormone receptor-positive HER2-negative (HR + HER-) advanced breast cancer after failure of treatment with letrozole or anastrozole. Results from the phase III BOLERO-2 trial demonstrated that everolimus and exemestane provide significant clinical benefit to patients with advanced HR + breast cancer. Although everolimus is generally well tolerated, as with most therapies administered in an advanced cancer setting, drug-related adverse events (AEs) inevitably occur^1^.

In the BOLERO-2 trial, hyperglycemia was listed among the most common grade 3 or 4 AEs (4% vs <1%, in the experimental and control arm, respectively)^1^. As a common toxicity associated with treatment with mTOR inhibitors, hyperglycemia is generally quite efficiently managed and pharmacologically controlled, with a low incidence of treatment discontinuation in the BOLERO-2 and subsequent studies of everolimus in breast cancer^1–5^.

Some considerations stem from the critical appraisal of the aforementioned literature. First, in none of these studies data on hyperglycemia were framed and interpreted in a wider context including measures of generalized and/or visceral obesity, e.g., body mass index (BMI) and waist circumference, respectively. A sound rationale to this approach may be found in the well established link between obesity and hyperglycemia and the combined effects of adiposity and hyperglycemia on patients’ important outcomes in HR + breast cancer^6–8^. In addition, previous experiences on the onset of selective toxicities when using targeted agents in the metastatic setting encourage evaluating hyperglycemia and, more generally, alterations of indicators of energetic metabolism in light of treatment efficacy^9,10^. Indeed, as a mTOR inhibitor, everolimus exerts its action on a target placed within a particularly complex network of pathways involved in the regulation of both the metabolic profile of the host and breast cancer development and progression. In these regards, the existence of regulatory loops between the insulin-like growth factor-1 receptor (IGF-1R)/insulin receptor substrate-1 (IRS-1) axis and the adenosine monophosphate–activated protein kinase (AMPK)/mTOR/protein-kinase 1 p70-S6 (S6K1) signaling cascade is well documented^11^. On this basis, it might be appropriate speculating that the efficacy of everolimus at a single patient level might be at least partly modulated by the dynamic equilibrium reached by these two axes following this drug administration. We thus speculated on the predictive role of anthropometric indicators of general adiposity, i.e., body mass index (BMI), and circulating biomarkers of glucose metabolism, i.e., fasting glucose, on treatment outcomes and verified the stated hypothesis in an observational, multicentre study of HR + HER2- advanced breast cancer patients treated with everolimus and exemestane in first and subsequent lines of therapy (the EverExt study).

## Methods

Six cancer institutions located in the Lazio region, Central Italy, contributed patients to our study. Prior to any study procedure, the study protocol was examined and approved by the Institutional Review Board at each of the participating centers, namely, the Regina Elena National Cancer Institute, Policlinico Sant’Andrea, Campus Bio-Medico University of Rome, ASL of Frosinone, G. d’Annunzio University, and San Giovanni Calibita Fatebenefratelli Hospital. A written informed consent was secured from each study participant. Our study was conducted in accordance with the Declaration of Helsinki.

Patients eligible to enter our study were postmenopausal women aged at least 18 years, with histologically confirmed, HR + HER2-advanced BC, and with an indication to treatment with everolimus and exemestane according to current indications and recommendations. At least one measurable or evaluable lesion had to be detectable. Further required criteria were an ECOG Performance Status (PS) of 0–2, adequate bone marrow, coagulation, liver, and renal function, ability to understand and willingness to sign a written informed consent. Exclusion criteria were previous diagnosis of other malignancies, except adequately treated non-melanoma skin cancer and/or curatively treated *in-situ* cancer of the cervix, symptomatic brain metastases, and any medical condition which might have interfered with safe participation in the study.

Eligible and consenting patients underwent assisted administration of questionnaires in course of face-to-face interviews to collect data on demographics and anthropometrics. FG assessment was performed in overnight fasting conditions both at baseline and in course of therapy. Data on the treatment in course of administration and related outcomes were prospectively collected, while records on the pathological features and previous treatment were retrieved by specifically trained research assistants under the supervision of the medical oncologists involved in the project.

To our study purposes, BMI was computed as the ratio between weight in kilograms and height in meters squared. Objective response (OR) was evaluated based on the Response Evaluation Criteria in Solid Tumours (RECIST) version 1.1. The best overall response (BR) was defined as the best response recorded from the start of the treatment under investigation until disease progression. Clinical benefit rate (CBR) was addressed as the percentage of patients with shrinking tumours or stable disease for at least 6 months. Progression free survival (PFS) was defined as the time elapsed between treatment start and interruption due to disease progression or death from any cause. Overall survival (OS) was defined as the time from the start of treatment to patient death from any cause. Toxicity was evaluated according to the National Cancer Institute Common Terminology Criteria for AE (NCI CTCAE) version 4.0.

Descriptive characteristics were analyzed for the overall study population and for all the variables of interest. We used means and standard deviations for continuous data and frequencies and percentage values for categorical data. Existing differences between mean values were evaluated using the Mann-Whitney or Kruskal-Wallis test, depending on the number of groups compared. Survival data were analyzed using the Kaplan-Meier method and differences among curves evaluated by log-rank test. The impact of anthropometric and metabolic biomarkers on survival was tested in Cox proportional Hazards models including variables testing significant at the univariate or selected on the basis of the relevance to the outcomes of interest. Statistical analyses were performed with SPSS statistical software version 20 (SPSS inc., Chicago IL, USA).

## Results

**Figure 1:**
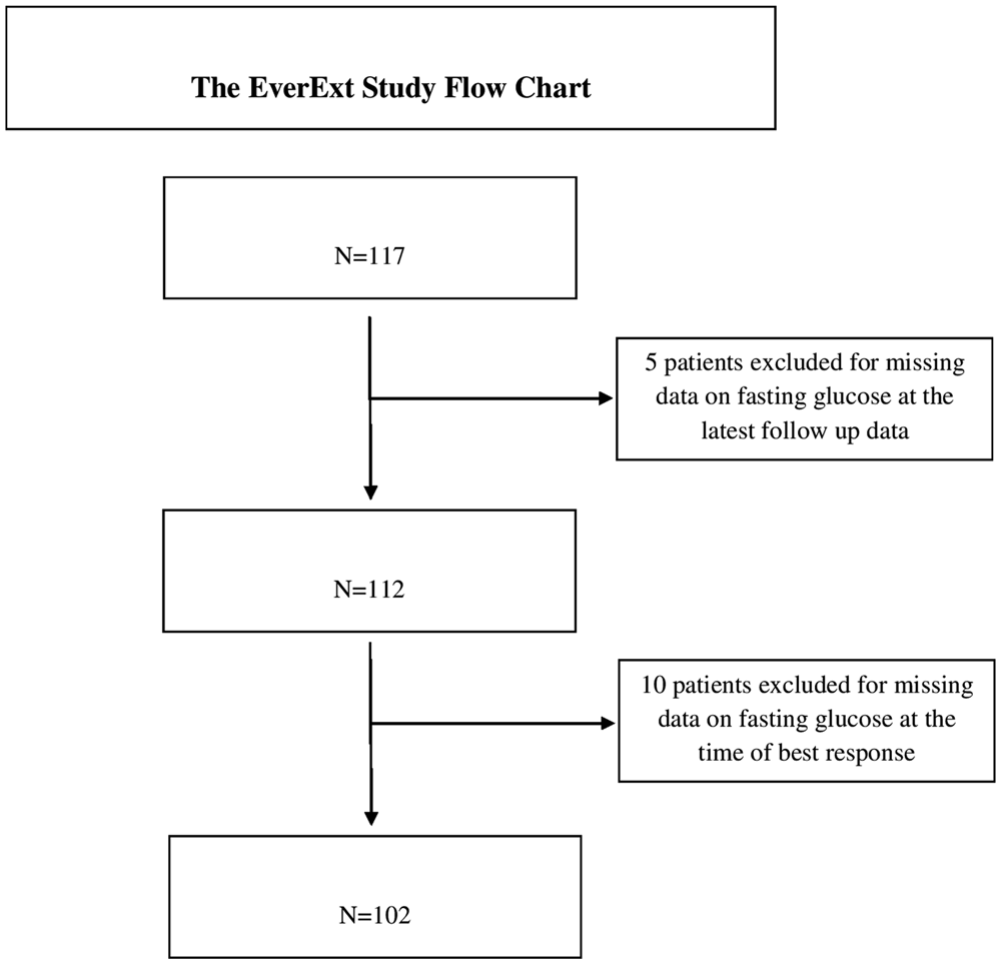
Study flow chart.

The flow chart of our study is shown in Fig. 1. At the six cancer institutes participating in our study, 117 patients were screened and judged eligible based on the inclusion criteria fully reported in the methods section. Among them, 15 patients were subsequently excluded due to missing data on FG in course of follow up. Thus, 102 patients contributed data to the final analysis.

### Patients’ descriptive characteristics

Our study participants’ characteristics are summarized in Table 1. In brief, mean age at diagnosis and baseline height, weight and BMI were 60.9 ± 10.1 years, 159.6 ± 6.2 cm, 69.1 ± 14.1 Kg, and 27.1 ± 5.6, respectively. Baseline values of FG were 101.2 ± 18.8, the mean number of cycles of everolimus and exemestane actually received was 9.0 ± 7.0, which in 30 (29.4%) patients were administered in first line. Overall, when comparing baseline values of FG and BMI with those assessed at the latest follow up for the entire study cohort, our data showed a significant trend towards increasing FG and decreasing BMI. Baseline vs final values of FG and BMI were 101.17 ± 18.83 vs 111.69 ± 28.82, p < 0.001 and 27.15 ± 5.58 vs 26.36 ± 5.59, p < 0.001, respectively. These differences remained significant when comparing means adjusted by number of cycles administered (99.93 ± 17.3 vs 107.3 ± 25.2, p < 0.001 and 27.26 ± 5.25 vs 26.43 ± 5.49, p < 0.001).

**Table 1:** Baseline characteristics of the study participants (N:102).

	mean ± SD
Age (years)	60.9 ± 10.1
Weight (Kg)	69.1 ± 14.1
Height (cm)	159.6 ± 6.2
BMI (Kg/m^2^)	27.1 ± 5.6
Fasting glucose (mg/dl)	101.2 ± 18.8
Number of cycles	9.0 ± 7.0
Line of therapy^*^	N (%)
1	30 (29.4)
>1	66 (64.7)

^*^6 missing.

### Fasting glucose and BMI at the time of disease reassessment and objective response evaluation

In Table 2, FG and BMI at the time of BR assessment were compared across groups defined upon BR and CBR. The mean values of FG and BMI were weighted against the number of cycles administered. Results showed significantly lower levels of FG at BR in group 1, i.e., in patients whose BR was either a CR/PR or SD, compared to group 2, i.e., patients whose disease progressed (108.9 ± 29.3 vs 122.6 ± 38.0, p < 0.001). Data also showed a suggestion for higher BMI values in the group of patients with CR/PR or SD (26.9 ± 5.3 vs 26.1 ± 6.4, p = 0.052). When distinguishing patients into three^3^ subgroups including women with CR/PR, cases with SD and those with progressive disease, results on FG were confirmed and those on BMI were statistically reinforced (supplementary Table 1). Consistently with what observed when addressing BR, a significantly higher number of patients in the lowest category of FG achieved CB compared to their counterpart (61 vs 41, p < 0.001), while there were no relevant differences in terms of BMI.

**Table 2:** Best response (BR) and Clinical Benefit (CB) by fasting glucose and BMI^1^ at the time of BR assessment (N:102).

Best Response			
	No PD2	PD2	Mann-Whitney
	N = 76	N = 26	
	Mean* ± SD	Mean* ± SD	p-value
Fasting glucose at BR	108.9 ± 29.3	122.6 ± 38.0	<0.001
BMI at BR	26.9 ± 5.3	26.1 ± 6.4	0.052
Clinical Benefit			
	Yes	No	Student’s T
	N = 41	N = 41	
	Mean* ± SD	Mean* ± SD	p-value
Fasting glucose at BR	108.2 ± 29.5	120.5 ± 33.0	<0.001
BMI at BR	26.8 ± 5.4	26.9 ± 5.4	0.895

^1^BMI: body mass index.

^2^PD: Disease Progression.

^*^Mean weighted by number of cycles administered.

### Survival analysis

**Figure 2:**
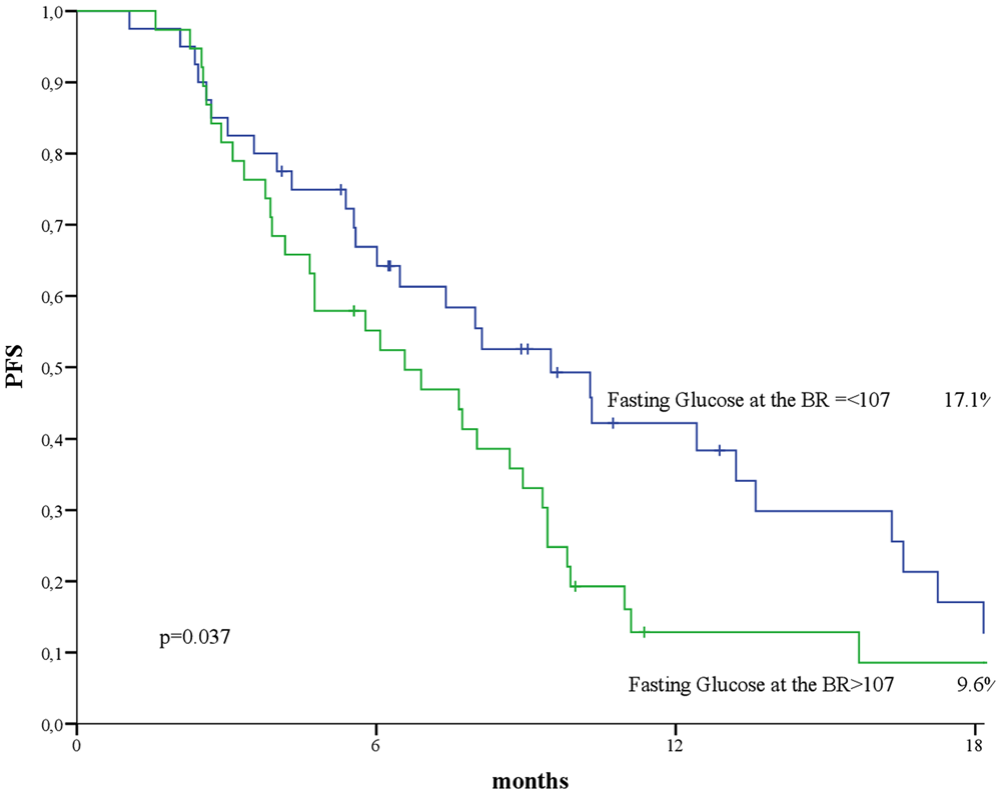
Progression free survival (PFS) by fasting glucose (FG) at best response (BR) (N:102).

Figure 2 illustrates PFS by levels of FG at BR. The cutoff of 107 represents the median value of FG at BR in the overall study population. At 18 months, 17.1% of patients whose FG at BR was in the lowest category were free from disease progression, compared with 8.6% of women in the highest FG category (p = 0.037), with median PFS and 95% CI being 9.50 (6.62–12.37) and 6.57 (3.85–9.29) months, respectively. This result was confirmed in uni- and multivariate analyses including BMI at BR and line of therapy (p = 0.040 and p = 0.049, for uni- and multivariate models, respectively) (Table 3

**Table 3:** Uni- and multivariate analysis of factors associated progression free survival (PFS) in the EverExt study.

	Univariate Analysis		Multivariate Analysis	
	HR (95%CI)	p-value	HR (95%CI)	p-value
Fasting Glucose at Best Response	1.70 (1.02–2.83)	0.040	1.69 (1.00–2.84)	0.049
BMI at Best Response	0.78 (0.47–1.28)	0.321	0.75 (0.45–1.25)	0.269
Line of Therapy (1 vs >1)	1.29 (0.74–2.24)	0.369	1.16 (0.66–2.04)	0.596

BMI: Body Mass Index.

HR: Hazard Ratio.

CI: Confidence Interval.

). Conversely, there was no impact of BMI at BR on the best objective response ever achieved (p = 0.169), with median PFS and 95% CI being 7.39 (3.42–11.36) and 9.33 (8.27–10.36) months in patients whose BMI at BR was in the lowest and highest category as defined upon the median value of 26.4, respectively. Neither FG nor BMI impacted OS (p = 0.89 and p = 0.17, respectively) (data available upon request).

### Toxicity

When addressing toxicity, data from 12 patients who dismissed treatment due to AE other than altered glucose levels were explored in light of changes in FG over the study period (final FG minus baseline FG/baseline FG). Changes in FG were significantly more pronounced in patients who quit treatment due to AEs other than hyperglycemia than in women who continued treatment (p = 0.014), while changes in BMI seemed not to affect discontinuation due to toxicity (p = 0.260).

## Discussion

In the proposed study, we assessed the predictive role of BMI and FG on efficacy and toxicity outcomes in 102 postmenopausal patients with HR + HER2−, metastatic BC treated with everolimus and exemestane at six cancer centers located in the Lazio region, centre Italy. We observed a significant trend towards increasing FG and decreasing BMI in the overall study population. We also found significant evidence of association of FG levels assessed at the time of BR recording with BR and CBR, with lower levels of FG being associated with better outcomes. There also was a suggestion for an association between higher BMI at BR and CR/PR or SD, which was statistically reinforced when comparing three^3^ distinct categories of OR, i.e., CR/PR vs SD vs PD, instead than two^2^, i.e., CR/PR/SD vs PD. or. We also found evidence in support of longer PFS in patients whose FG levels were in the lowest category defined upon the median FG value for the overall study population. This result was confirmed in Cox models including line of therapy and BMI. None of the exposures of interest seemed to affect OS. In addition, changes in FG over the course of treatment were significantly more pronounced in patients who suspended treatment due to toxicity other than hyperglycemia.

Over the past few years, the evidence concerning the efficacy and safety of the treatment of interest has immensely grown with large and consistent data being provided from both randomized clinical trials and real practice^1–5,12^. Data from the BOLERO-2 phase III clinical trial and the BALLET expanded access program (EAP) have been particularly informative on the efficacy and feasibility of this treatment, respectively^1,5^. With specific regards to the exposures of interest, i.e., FG and BMI, we observed significant evidence of increasing FG and decreasing BMI in the overall population. The closest evidence which may attain to our results is concerning the incidence and time course of hyperglycemia in trials of everolimus and exemestane in breast cancer. In the BOLERO-2 trial, hyperglycemia was defined based on the criteria stated by the American Diabetes Association (ADA), namely, fasting glucose levels >130 mg/dL or postprandial levels >180 mg/dL. Thirteen^13^ vs 2 events were recorded in the experimental and control arm, respectively. Four percent (4%) of these AE were graded 3 or 4 in the patients treated with EverExt, while the reported percentage was inferior to 1(˃1%) in patients having received exemestane and placebo^1–13^. When focusing on elderly patients enrolled in BOLERO-2 trial, hyperglycemia was again listed among the most commonly observed grade 3 or 4 AEs (≥5%) in patients from the experimental arm, along with stomatitis, fatigue, dyspnea, anemia, and increased gamma-glutamyltransferase levels, which were similar across age groups^2^. Based on the data published subsequently by Rugo and co-authors, 9% of patients in the experimental arm and 10% in the control arm exhibited diabetes mellitus or impaired glucose tolerance on study entrance. During the study, 11% of the patients in the experimental arm developed grade ≥2 hyperglycemia or newly-onset diabetes mellitus compared with 2% of patients in the control arm. The incidence of all-grade hyperglycemia and new-onset diabetes mellitus was higher in the experimental group than in the control arm (16% versus 3%, respectively), with grade 3 or 4 events being observed in 6% and 1% of patients, respectively^5^. Ravaud and colleagues carried out an individual patient data meta-analysis including data from five phase 2/3 studies of patients with solid tumours treated with everolimus not including breast cancer. Overall, 945 and 938 patients were evaluable for efficacy and safety, respectively. Hyperglicemia occurring in course of treatment and lipid alterations were grouped within the same category and globally referred to as “metabolic events”. Overall, results from this meta-analysis support the association between higher everolimus exposure and more pronounced tumour reduction, longer PFS and increased risk of high-grade pulmonary, stomatitis and metabolic events^14^. The authors also openly refer to the paucity of data currently available from the literature to support or adverse the hypothesis of a relationship between AEs and efficacy of mTOR inhibitors. Supportive evidence is provided by a retrospective analysis of 46 patients with renal cell carcinoma (RCC) treated with everolimus (N: 25) or temsirolimus (N: 21). In these patients, a SD was reported in 85.7% of the patients who experienced pneumonitis (N:14) versus 43.8% of those who did not (N:32)^15^. A larger retrospective analysis including data of 310 patients with RCC having received a mTOR inhibitor showed that non-infectious pneumonitis had a predictive role on OS, with better outcome in patients having developed this AE^16^. In addition, data from the meta-analysis from Ravaud and co-authors seem to be consistent with results from these latter studies, in that everolimus exposure was significantly associated with high grade pulmonary, metabolic and stomatitis AEs and reduced tumour size^14^. Pantano and colleagues have recently addressed the changes of lipid metabolism in a case series of 117 patients with advanced renal cell carcinoma treated with everolimus. Their results showed a predictive role of the combined rising of cholesterol and tryglicerides on treatment outcomes, with median time to progression being significantly longer in the subgroup of patients who showed this metabolic alteration in course of treatment compared to those who did not (10.9 vs 5.9 months, respectively, p = 0.003)^17^.

We also observed evidence of association of FG levels assessed at the time of BR recording with BR and CBR, with lower levels of FG being associated with better outcomes. In addition, longer PFS was associated with lower FG levels in uni- and multivariate analysis. Our findings concerning the predictive role of FG and BMI on treatment outcomes in the population of interest are novel. Such novelty can be traced both in the hypothesis stated and results obtained. Indeed, previous studies of everolimus in breast cancer have repeatedly addressed alterations in circulating levels of FG within the context of fasting or postprandial hyperglycemia. However, the statistics provided are essentially descriptive and no prior attempts to investigate the potential impact of such abnormalities on treatment outcomes have been pursued. In addition, our data on FG were integrated by the repeated collection of anthropometrics, which were also evaluated in ad hoc analysis and included in multivariate models of FG. Data were collected prospectively by specifically trained research personnel with an active role in clinical practice. This increases our confidence in our data quality and overall reliability of this study results^18,19^.

Our study also has some limitations. Among them, the observational nature of our findings, which invites caution in results interpretation and imposes limits to their generalizability. This is a common features of several studies carried out by our group, which may anyway be outweighed by their potentialities in terms of their “hypothesis generating” potentials^10–23^. We also acknowledge the restricted number of exposures evaluated, i.e., fasting glucose and BMI only, as predictors of treatment outcomes in our study population. We are aware of the need for a more comprehensive evaluation which including, though not limited to, the systematic estimation of parameters related to the regional fat tissue representation, e.g., arms’ fat, legs’ fat and trunk fat. We could not find any evidence in support of an association between the exposures of interest and OS. This may at least partly be dependent on the relatively short length of follow up, with the median and range being 12 (1.0–4.1) months. In the hypothesis of future studies with a longer period of observation, the potential role of lines of therapy administered following everolimus exemestane should be considered in analysis of OS, along with punctual data on the number of lines and agents administered. In our study, FG measurements were not centralized at the institutional labs of the participating centres. When conceiving the study, we relied on the use of external laboratories for the repeated assessment of FG of the consenting participants. Notwithstanding the superior quality of any form of centralized assessment, our choice helped avoid excessive burden for the institutional labs both in terms of time and economic resources, which, given the spontaneous nature of our study, were limited. At the same time, our decision helped control the number of visits at the participating centres, thus maximizing the rate of acceptance and increasing our study feasibility both at an institutional and patient level.

## Conclusions

In summary, we conducted an observational, prospectively designed study of 102 metastatic, HR + HER2−, metastatic BC patients treated with everolimus and exemestane in first and subsequent lines of therapy at six Italian cancer centres. Data analysis revealed a predictive role of FG and BMI on treatment outcomes, with lower FG levels recorded at the time of BR being associated with better OR, CBR and longer PFS. Some evidence of association of lower BMI at BR with better outcomes was also observed. Similarly, altered FG seemed to be more common in patients who dismissed treatment due to AEs other than hyperglycemia. Whether or not alterations in energetic metabolism linked to the use of everolimus may significantly impact this drug efficacy and its safety profile remains an open question. Evidence from the EvereExt study is suggestive but in need of extensive and multidisciplinary integrations. Future studies within this pipeline have been planned, with a specific focus on the molecular mechanisms underlying the phenomena observed. We will considerably expand the spectrum of circulating biomarkers and include FG, insulin, and insulin like growth factor I, and indicators of general and visceral adiposity, i.e., BMI and waist circumference, and measures of regional fat distribution, e.g., arms’ fat, legs’ fat and trunk fat In addition, collection of genomics and trascriptomics data on the components of key signaling pathways with a mutual role in breast oncogenesis and glucose homeostasis, e.g. PI3K/AKT and JUN/MAPK will be of pivotal importance in clarifying the underlying molecular mechanisms^24,25^.

## Methods

Six cancer institutions located in the Lazio region, Central Italy, contributed patients to our study. Prior to any study procedure, the study protocol was examined and approved by the Institutional Review Board at each of the participating centers. Patients eligible to enter our study were postmenopausal women aged at least 18 years, with histologically confirmed, HR + HER2− advanced BC, and with an indication to treatment with everolimus and exemestane according to current indications and recommendations. At least one measurable or evaluable lesion had to be detectable. Further required criteria were an ECOG Performance Status (PS) of 0–2, adequate bone marrow, coagulation, liver, and renal function, ability to understand and willingness to sign a written informed consent. Exclusion criteria were previous diagnosis of other malignancies, except adequately treated non-melanoma skin cancer and/or curatively treated *in-situ* cancer of the cervix, symptomatic brain metastases, and any medical condition which might have interfered with safe participation in the study.

Eligible and consenting patients underwent assisted administration of questionnaires in course of face-to-face interviews to collect data on demographics and anthropometrics. FG assessment was performed in overnight fasting conditions both at baseline and in course of therapy. Data on the treatment in course of administration and related outcomes were prospectively collected, while records on the pathological features and previous treatment were retrieved by specifically trained research assistants under the supervision of the medical oncologists involved in the project.

To our study purposes, BMI was computed as the ratio between weight in kilograms and height in meters squared. Objective response (OR) was evaluated based on the Response Evaluation Criteria in Solid Tumours (RECIST) version 1.1. The best overall response (BR) was defined as the best response recorded from the start of the treatment under investigation until disease progression. Clinical benefit rate (CBR) was addressed as the percentage of patients with shrinking tumours or stable disease for at least 6 months. Progression free survival (PFS) was defined as the time elapsed between treatment start and interruption due to disease progression or death from any cause. Overall survival (OS) was defined as the time from the start of treatment to patient death from any cause. Toxicity was evaluated according to the National Cancer Institute Common Terminology Criteria for AE (NCI CTCAE) version 4.0.

Descriptive characteristics were analyzed for the overall study population and for all the variables of interest. We used means and standard deviations for continuous data and frequencies and percentage values for categorical data. Existing differences between mean values were evaluated using the Mann-Whitney or Kruskal-Wallis test, depending on the number of groups compared. Survival data were analyzed using the Kaplan-Meier method and differences among curves evaluated by log-rank test. The impact of anthropometric and metabolic biomarkers on survival was tested in Cox proportional Hazards models including variables testing significant at the univariate or selected on the basis of the relevance to the outcomes of interest. Statistical analyses were performed with SPSS statistical software version 20 (SPSS inc., Chicago IL, USA).

## Declarations

### Ethics approval and consent to participate

The study protocol was evaluated for approval by the Institutional Review Board of the coordinating centre, i.e., the Regina Elena National Cancer Institute of Rome, on the 15^th^ July 2014. The approved documents were then transmitted for further examination to each of the participating centers. Written consents were secured from the study participants.

### The study protocol

The code assigned to our study protocol at the time of evaluation and approval by the Institutional Review Board is as follows: CE/542/14. The study protocol will be made available upon request.

### Consent for publication

Individual patient data were anonymized prior to inclusion in our analyses. The institutional consent form will be made available upon request at any stage (eventually including publication).

### Availability of data and material

The datasets during and/or analyses during the current study available from the corresponding author on reasonable request.

## Electronic supplementary material

Supplementary table 1
