# Supplementary material for: Fasting glucose and body mass index as predictors of activity in breast cancer patients treated with everolimus-exemestane: The EverExt study
Source: Sci Rep. 2017 Sep 6;7:10597. doi: 10.1038/s41598-017-10061-2 (PMC5587713; doi:10.1038/s41598-017-10061-2)
Supplement: Supplementary file 1 — Supplementary table 1 [file 41598_2017_10061_MOESM1_ESM.pdf]

Title: Fasting glucose and body mass index as predictors of activity in breast cancer patients treated with everolimus-exemestane: The EverExt study.

Authors: Pizzuti L, Marchetti P, Natoli C, Gamucci T, Santini D, Scinto AF, Iezzi L, Mentuccia L, D'Onofrio L, Botticelli A, Moscetti L, Sperati F, Botti C, Ferranti F, Buglioni S, Sanguineti G, Di Filippo S, Di Lauro L, Sergi D, Catenaro T, Tomao S, Giordano A, Maugeri-Saccà M, Barba M*, and Vici P.

*corresponding author

Supplementary table 1. Objective response (OR) by fasting glucose and BMI[1] at the time of disease re-assessment (N:102).

	Objective Response<br>Group 1<br>CR/PR<br>N=37<br>Mean*±SD	Group 2<br>SD<br>N=39<br>Mean*±SD	Group 3<br>PD<br>N=26<br>Mean*±SD	Kruskal-Wallis<br>p-value
**Fasting glucose at BR**	111.5±31.0	105.8±26.8	122.6±38.0	<0.001
**BMI at BR**	26.7±5.9	27.1±4.5	26.1±6.4	0.005

[1]BMI: body mass index

[2]PD: Disease Progression

* mean weighted by number of cycle

** mean weighted by number of cycle computed in patients without PD

*** mean weighted by number of cycle computed in patients with PD
